# miR-127 enhances myogenic cell differentiation by targeting *S1PR3*

**DOI:** 10.1038/cddis.2017.128

**Published:** 2017-03-30

**Authors:** Lili Zhai, Rimao Wu, Wanhong Han, Yong Zhang, Dahai Zhu

**Affiliations:** 1 The State Key Laboratory of Medical Molecular Biology, Institute of Basic Medical Sciences, Chinese Academy of Medical Sciences and School of Basic Medicine, Peking Union Medical College, 5 Dong Dan San Tiao, Beijing 100005, PR China

## Abstract

MicroRNAs (miRNAs) have recently been implicated in muscle stem cell function. miR-127 is known to be predominantly expressed in skeletal muscle, but its roles in myogenic differentiation and muscle regeneration are unknown. Here, we show that miR-127 is upregulated during C2C12 and satellite cell (SC) differentiation and, by establishing C2C12 cells stably expressing miR-127, demonstrate that overexpression of miR-127 in C2C12 cells enhances myogenic cell differentiation. To investigate the function of miR-127 during muscle development and regeneration *in vivo*, we generated miR-127 transgenic mice. These mice exhibited remarkably accelerated muscle regeneration compared with wild-type mice by promoting SC differentiation. Mechanistically, we demonstrated that the gene encoding sphingosine-1-phosphate receptor 3 (S1PR3), a G-protein-coupled receptor for sphingosine-1-phosphate, is a target of miR-127 required for its function in promoting myogenic cell differentiation. Importantly, overexpression of miR-127 in muscular dystrophy model *mdx* mice considerably ameliorated the disease phenotype. Thus, our findings suggest that miR-127 may serve as a potential therapeutic target for the treatment of skeletal muscle disease in humans.

Skeletal muscle regeneration relies on a small population of stem cells, termed satellite cells (SCs), that reside beneath the basal lamina of myofibers.^1, 2^ SCs are normally quiescent; in response to stress or injury, however, they become activated and proliferate, differentiate, and fuse into multinucleated myotubes.^3, 4^ Abnormalities in SC activation, proliferation, or differentiation result in skeletal muscle dysfunction during regeneration, leading to the development of muscle disease.^5^

MicroRNAs (miRNAs) are a class of ~22-nucleotide-long noncoding RNAs that regulate gene expression at the post-transcriptional level. miRNAs help regulate many different processes, including cell-fate determination, proliferation, differentiation, and apoptosis, during normal development and in disease.^6, 7, 8, 9, 10^ MyoD-Cre- or Pax7-Cre-mediated knockout of Dicer, an RNase III endonuclease responsible for miRNA maturation, in mouse skeletal muscle revealed that miRNAs are required for muscle development and SC functions.^11, 12^ In particular, the miRNAs, miR-1 and miR-133, are induced during skeletal muscle differentiation and have regulatory roles in myoblast proliferation and differentiation.^13^ Moreover, miR-1 and miR-206 regulate SC differentiation by repressing the transcription factor, *Pax7* (paired box 7).^14^ In addition, miR-27a, which is expressed in differentiating skeletal muscle of the embryonic myotome and in activated SCs of adult muscles, promotes SC differentiation by targeting *Pax3*.^15^ Recently, we reported that miR-431 is predominantly expressed in myogenic lineage cells and regulates SC heterogeneity, and further showed that miR-431 transgenic mice harbor a larger Pax7^Lo^ SC sub-population and exhibit accelerated muscle regeneration.^16^ Interestingly, aberrant miRNA expression has also been reported in human muscular dystrophy patients.^17, 18^ In this context, miR-206 and miR-431 have been demonstrated to improve symptoms in the *mdx* mouse model of muscular dystrophy.^16, 19^

miR-127, located within an miRNA cluster in the Dlk1-Dio3 region of both mouse and human genomes,^20, 21^ has been implicated in the development of breast cancer, hepatocellular cancer, glioblastoma, and lung carcinomas.^22, 23, 24, 25^ Interestingly, several miRNAs in this cluster, including miR-431, miR-127, miR-432, miR-433, miR-434, and miR-136, have been reported to be predominantly expressed in skeletal muscle and brain tissues.^16, 26, 27^ Our group and others recently demonstrated that miR-431 has an important role in muscle stem cell function and muscle regeneration,^16, 28^ and it is conceivable that miR-127 might be involved in muscle development or SC functions during postnatal myogenesis and regeneration.

Here, we show that miR-127 is upregulated during SC and C2C12 cell differentiation, and that overexpression of miR-127 significantly potentiates myogenic differentiation. Using miR-127 transgenic mice, we demonstrate that miR-127 enhances skeletal muscle regeneration and ameliorates muscular dystrophy in *mdx* mice by promoting SC differentiation. We further identify the *S1PR3* (sphingosine-1-phosphate receptor 3) gene as a direct target of miR-127, and show that it is mechanistically involved in miR-127-mediated SC differentiation and muscle regeneration.

## Results

### miR-127 enhances C2C12 cell differentiation

**Figure 1:**
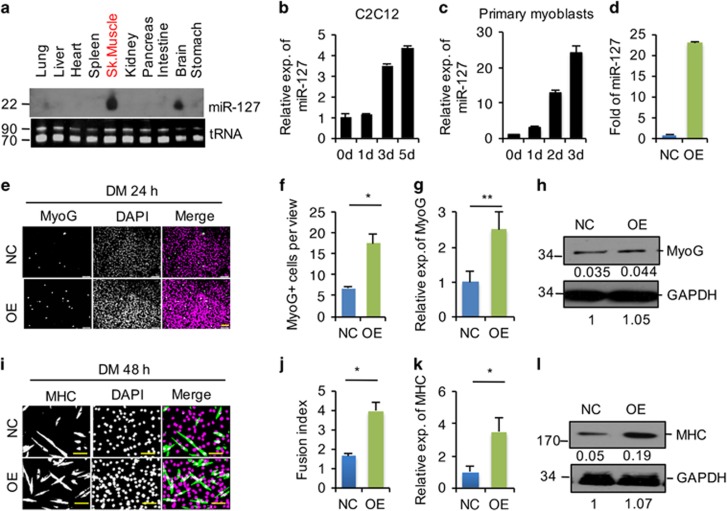
miR-127 enhances C2C12 cell differentiation. (**a**) Detection of the mature form of miR-127-3p by Northern blotting in the indicated tissues from 3-week-old mice. Transfer RNA (tRNA) was used as a loading control. (**b** and **c**) Quantification of miR-127-3p expression in C2C12 cells (**b**) and primary myoblasts (**c**) by quantitative real-time polymerase chain reaction (qRT-PCR) at the indicated days during differentiation. The data were normalized using U6. *miR-127* expression levels were further normalized to the expression level of 0 days, defined as 1. (**d**) Fold overexpression of *miR-127-3p* in C2C12 cells, quantified by qRT-PCR. OE, C2C12 cells infected with miR-127-expressing lentivirus; NC, C2C12 cells infected with control lentivirus. The data were normalized using U6. *miR-127* expression levels were further normalized to the expression level of NC, defined as 1. (**e**) Immunostaining for MyoG (green) and DAPI (magenta) after 24 h of culture in DM. Scale bars, 50 *μ*m. (**f**) Quantification of MyoG^+^ cells presented in panel e. The data are representative of three independent experiments. For each experiment, 10 representative views were analyzed, and data are presented as positive cell numbers per view. Statistical analysis was performed with nonparametric tests. (**g**) Quantification of *MyoG* mRNA levels by qRT-PCR in NC and miR-127 OE cells, differentiated as in panel e. The data were normalized using GAPDH. *MyoG* expression levels were further normalized to the expression level of NC, defined as 1. Statistical analysis was performed with nonparametric tests. (**h**) Western blot analysis of MyoG protein levels in NC and miR-127 OE cells, differentiated as in panel e. GAPDH was used as a loading control. The numbers below each blot were the relative quantification of band intensity determined by Image J. (**i**) Immunostaining for MHC (green) and DAPI (magenta) after 48 h of culture in DM. Scale bars, 50 *μ*m. (**j**) Fusion index of the differentiated cells presented in panel I. The fusion index is calculated as the percentage of total nuclei present in multinucleated myotubes. The data are representative of three independent experiments. For each experiment, 10 representative views were analyzed. (**k**) Quantification of *MHC* mRNA levels by qRT-PCR in NC and miR-127 OE cells, differentiated as in panel I. The data were normalized using GAPDH. *MHC* expression levels were further normalized to the expression level of NC, defined as 1. (**l**) Western blot analysis of MHC protein levels in NC and miR-127 OE cells, differentiated as in panel (**i**). GAPDH was used as a loading control. The numbers below each blot were the relative quantification of band intensity determined by Image J. Value in graphs represent means±S.E. of three independent experiments. *P<0.05; **P<0.01

As shown in Figure 1a and consistent with published data,^26, 27^ we found that miR-127 is predominantly expressed in the skeletal muscle and the brain. Interestingly, miR-127 was significantly upregulated in response to the induction of myogenic differentiation in both the C2C12 mouse myoblast cell line (Figure 1b and Supplementary Figure S1) and primary mouse myoblasts (Figure 1c) as reported very recently,^27^ indicating a functional role of miR-127 in regulating myogenic cell differentiation.

To investigate directly the impact of miR-127 on myogenic cell differentiation, we established C2C12 cell lines stably overexpressing (OE) miR-127 or the empty vector as a negative control (NC). The expression of miR-127 was ~25-fold higher in miR-127 OE cells compared with that in NC cells (Figure 1d). Immunostaining for the early myogenic differentiation marker myogenin (MyoG) revealed significantly increased the number of differentiating cells in miR-127 OE cultures than in NC cultures following induction of differentiation (Figures 1e and f). Consistent with the MyoG staining results, levels of MyoG mRNA (Figure 1g) and protein (Figure 1h) were also significantly increased in miR-127 OE cells relative to NC cells. The ability of miR-127 to stimulate C2C12 cell differentiation is further supported by the observed increase in myosin heavy chain (MHC) immunoreactivity (Figure 1i) and fusion index (Figure 1j), and higher levels of *MHC* mRNA (Figure 1k) and protein (Figure 1l) in miR-127 OE cells. These results indicate that miR-127 significantly potentiates myogenic cell differentiation *in vitro*.

### miR-127 accelerates skeletal muscle regeneration by promoting SC differentiation

**Figure 2:**
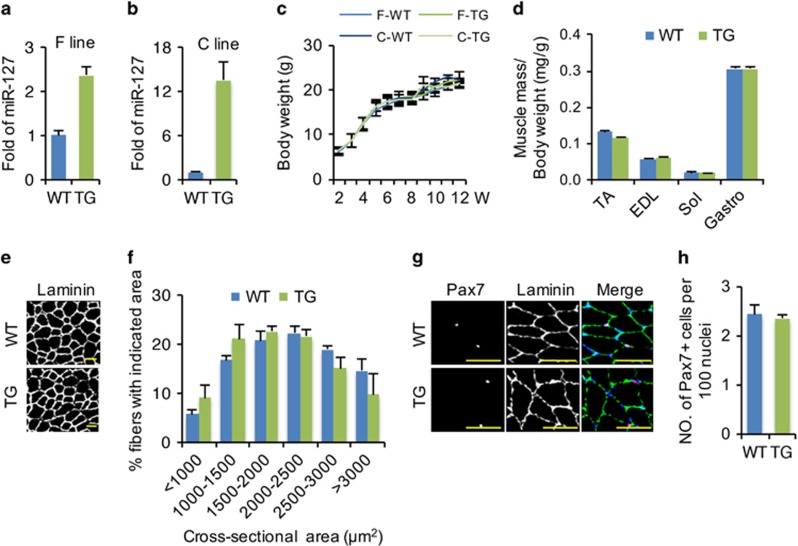
No significant difference between WT and miR-127 TG mice during normal development. (**a** and **b**) Fold overexpression of *miR-127-3p* in the skeletal muscle of the TG mouse F line (**a**) and C line (**b**) relative to WT littermates. The data were normalized using U6. *miR-127* expression levels were further normalized to the expression level of WT, defined as 1. (**c**) Growth curves for WT and miR-127 TG mice, showing plots of body weights measured weekly (*n*=8 mice per group). (**d**) The ratio of individual muscle mass (mg) to body weight (**g**) in 8-week-old WT and miR-127 TG mice for various muscle tissues. (**e**) Immunostaining for laminin in TA muscle from 8-week-old WT and miR-127 TG mice. Scale bars, 100 *μ*m. (**f**) Cross-sectional areas of myofibers in TA muscles from WT and miR-127 TG mice (*n*=5 mice per group). (**g**) Immunostaining for Pax7 (magenta), laminin (green) and DAPI (blue) in TA muscle from 8-week-old WT and miR-127 TG mice. Scale bars, 50 *μ*m. (**h**) Quantification of Pax7^+^ cell numbers per 100 nuclei in TA muscle from 8-week-old WT and miR-127 TG mice (*n*=8 mice per group). Values in graphs represent means±S.E. EDL, extensor digitorum longus; Gastro, gastrocnemius; Sol, soleus; TA, tibialis anterior

The ability of miR-127 to enhance C2C12 cell differentiation *in vitro* prompted us to investigate its functional role in regulating skeletal muscle development and regeneration *in vivo*. For this purpose, we generated two miR-127 TG mice lines (C line and F line), in which miR-127 overexpression was driven by the *β*-actin promoter.^29, 30^ Although *miR-127* expression in the skeletal muscle was increased 2.5-fold in the F line (Figure 2a) and 14-fold in the C line (Figure 2b), we did not observe significant difference in bodyweight (Figure 2c), muscle mass (Figure 2d) or fiber size (Figures 2e and f) between either line miR-127 TG mice and wild-type (WT) mice at the indicated ages. Additionally, we found no significant difference in Pax7-positive (Pax7^+^) SC numbers between WT and miR-127 TG mice (Figures 2g and h). The phenotypes of the two miR-127 TG mouse lines were similar; thus, we selected the F line for detailed characterization.

**Figure 3:**
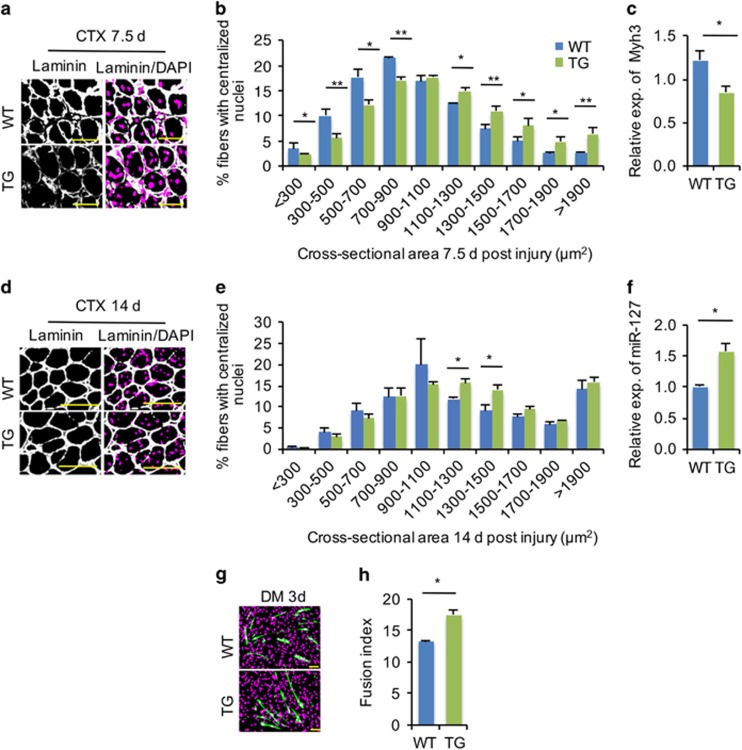
miR-127 accelerates skeletal muscle regeneration by promoting SC differentiation. (**a**) Immunostaining for laminin (green) and DAPI (magenta) in TA muscles obtained from WT and miR-127 TG mice 7.5 days after CTX-induced injury. Scale bars, 50 *μ*m. (**b**) Cross-sectional areas of regenerated myofibers containing centralized nuclei were counted in WT and miR-127 TG TA muscle 7.5 days after CTX injury. (**c**) Quantitative real-time polymerase chain reaction (qRT-PCR) analysis of *Myh3* gene expression at 7.5 days postinjury. (**d**) Immunostaining for laminin (green) and DAPI (magenta) in TA muscles obtained from WT and miR-127 TG mice 14 days after CTX-induced injury. Scale bars, 50 *μ*m. (**e**) Cross-sectional areas of regenerated myofibers and myofibers containing centralized nuclei were counted in WT and miR-127 TG TA muscle 14 days after CTX injury. (**f**) qRT-PCR analysis of fold overexpression of *miR-127-3p* in primary myoblasts from WT and miR-127 TG mice. The data were normalized using U6. *miR-127* expression levels were further normalized to the expression level of WT, defined as 1. (**g**) Immunostaining for MHC (green) and DAPI (magenta) in differentiated primary myoblasts isolated from hindlimb muscles of miR-127 TG mice and WT littermates after 3 days of culture in DM. Scale bars, 50 *μ*m. (**h**) Fusion indices were calculated based on MHC-stained cells presented in panel g. Value in graphs represents means±S.E. of at least three independent experiments. *P<0.05; **P<0.01

Next, we assessed the functional role of miR-127 in mediating skeletal muscle regeneration. To this end, we injured tibialis anterior (TA) muscles from miR-127 TG and WT mice using cardiotoxin (CTX) as described previously.^16^ To assess directly the functional role of miR-127 in regulating SC differentiation during regeneration, we measured the size of regenerating myofibers 7.5 days postinjury. As shown in Figures 3a and b, regenerating myofibers, characterized by centralized nuclei, had larger cross-sectional areas in miR-127 TG mice than in WT controls. Moreover, the expression of embryonic MHC (Myh3) was less abundant in the damaged muscles of TG mice than in WT mice at 7.5 days postinjury (Figure 3c), indicating that newly formed myofibers were bigger in TG mice than in WT controls. The more rapid formation of regenerative myofibers was further corroborated at 14 days postinjury (Figures 3d and e). Collectively, this morphological and molecular evidence revealed that myogenic differentiation was significantly accelerated in miR-127 TG mice undergoing muscle regeneration.

The functional role of miR-127 in promoting myogenic cell differentiation was further validated using primary myoblasts isolated from hindlimb skeletal muscles of miR-127 TG and WT mice. As expected, *miR-127* expression was higher in primary myoblasts isolated from miR-127 TG mice than in those from WT mice (Figure 3f). The isolated cells were induced to differentiate in low-serum medium for 3 days, after which myogenic differentiation was characterized by examining the fusion index. We found significantly increased fusion index (Figures 3g and h) in primary myoblasts from miR-127 TG mice compared with cells from WT controls. Taken together, these findings reveal a significant role of miR-127 in promoting myogenic differentiation, both *in vitro* and *in vivo*.

### miR-127 augments myogenic differentiation by targeting S1PR3

**Figure 4:**
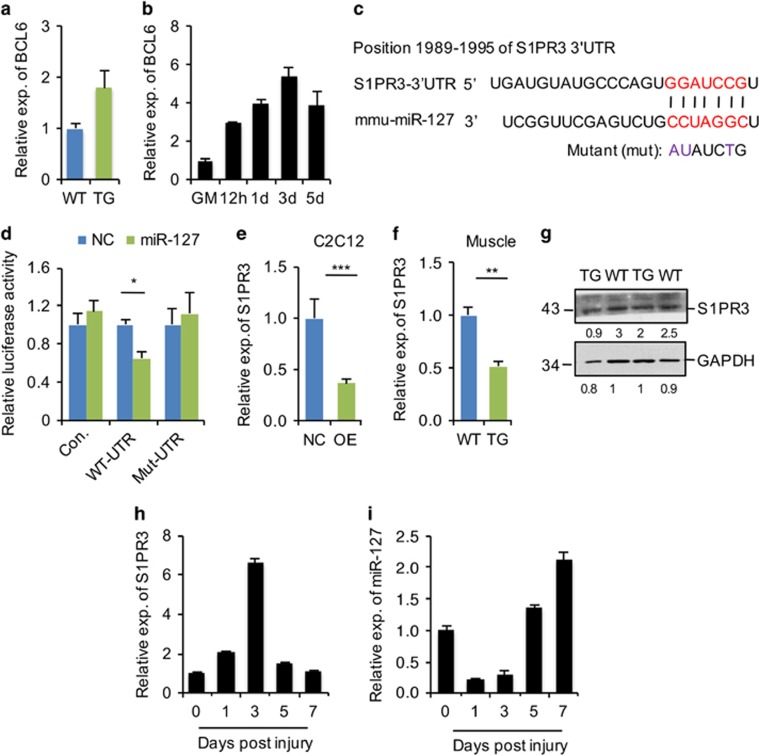
*S1PR3* is a target of miR-127 both *in vitro* and *in vivo*. (**a**) Quantitative real-time polymerase chain reaction (qRT-PCR) analysis of *BCL6* gene expression in TA muscle from 8-week-old WT and miR-127 TG mice. The data were normalized using *GAPDH*. *BCL6* expression levels were further normalized to the expression level of WT, defined as 1. (**b**) Quantification of *BCL6* mRNA in C2C12 cells grown in growth media (GM) and the indicated days during differentiation, determined by qRT-PCR. The data were normalized using *GAPDH*. *BCL6* expression levels were further normalized to the expression level of GM, defined as 1. (**c**) Sequence alignment shows the target sites of miR-127 in the 3′-UTR of mouse *S1PR3*, as predicted by miRWalk. A mutation in the seed matches is indicated by ‘mut'. (**d**) miR-127 directly repressed WT *S1PR3* 3′-UTR in luciferase assays in HEK293 cells, and the repression was abolished by mutation of the miR-127 binding site in the *S1PR3* 3′-UTR. The values are means±S.E. from three independent experiments. (**e**) Quantification of *S1PR3* mRNA levels in miR-127-OE and control (NC) C2C12 cells by qRT-PCR. The data are a representative of three independent experiments, each performed in triplicate. The data were normalized using *GAPDH*. *S1PR3* expression levels were further normalized to the expression level of NC, defined as 1. (**f**) *S1PR3* mRNA levels in TA muscle from 8-week-old WT and miR-127 TG mice, determined by qRT-PCR (*n*=5 mice per genotype). The data were normalized using *GAPDH*. *S1PR3* expression levels were further normalized to the expression level of WT, defined as 1. (**g**) Western blot analysis of S1PR3 protein in TA muscles of 8-week-old WT and miR-127 TG mice. The numbers below each blot represent quantified signal intensities of individual bands. (**h**) qRT-PCR analysis of *S1PR3* in TA muscle at the indicated days after CTX-induced injury (*n*=3 mice per genotype). The data were normalized using *GAPDH*. *S1PR3* expression levels were further normalized to the expression level of 0 days, defined as 1. (**i**) qRT-PCR analysis of *miR-127-3p* in the same samples described in panel h. The data were normalized using U6. *miR-127* expression levels were further normalized to the expression level of 0 days, defined as 1. Values in graphs represent means±S.E. *P<0.05; **P<0.01; and ***P<0.001

Next, we investigated the molecular mechanism underlying the function of miR-127 in promoting myogenic cell differentiation by identifying its targets. It has been reported that BCL6 (B-cell CLL/lymphoma 6) is an miR-127 target in cancer cells^13, 23, 31^ whose expression is upregulated during myogenic differentiation in C2C12 cells,^32^ suggesting that miR-127 might promote myogenic differentiation by targeting BCL6. However, our data did not support this possibility, as evidenced by the observations that *BCL6* mRNA levels were not reduced in miR-127-OE muscle (Figure 4a) and expression of the *BCL6* gene was upregulated during C2C12 cell differentiation (Figure 4b), an expression pattern similar to that of miR-127 (Figure 1b). We then further predicted functional targets of miR-127 using computational and bioinformatics-based approaches. Among the predicted miR-127 targets, *S1PR3* (sphingosine-1-phosphate receptor 3) seemed particularly interesting because of its previously reported roles in muscle differentiation and regeneration.^11, 33^

*S1PR3*, which contains a putative miR-127 binding site in its 3′-untranslated region (3′-UTR) (Figure 4c), was ranked 75 out of more than 10 000 targets of miR-127 predicted by the analysis tool, miRWalk. To experimentally validate that *S1PR3* is a target of miR-127, we generated luciferase reporter constructs carrying the 3′-UTR sequence of WT *S1PR3* (WT-UTR) and a mutant form (mut-3′-UTR) harboring substitutions in the miR-127 binding sites in the 3′-UTR. As shown in Figure 4d, co-transfection of miR-127 mimics decreased the luciferase activity of WT-UTR, but not that of mut-3′-UTR. The control pGL-3 construct was insensitive to miR-127. These results indicate that miR-127 directly targets *S1PR3*.

To further establish a functional link between miR-127 and *S1PR3*, we first examined the expression of endogenous *S1PR3* in miR-127 OE C2C12 cells and in thr skeletal muscle of miR-127 TG mice. We found that *S1PR3* mRNA levels were decreased in miR-127 OE cells (Figure 4e). Consistent with this, both mRNA (Figure 4f) and protein (Figure 4g) levels of S1PR3 were also lower in the skeletal muscle of miR-127 TG mice than WT mice, indicating that miR-127 directly targets SIPR3 both *in vitro* and *in vivo*.

**Figure 5:**
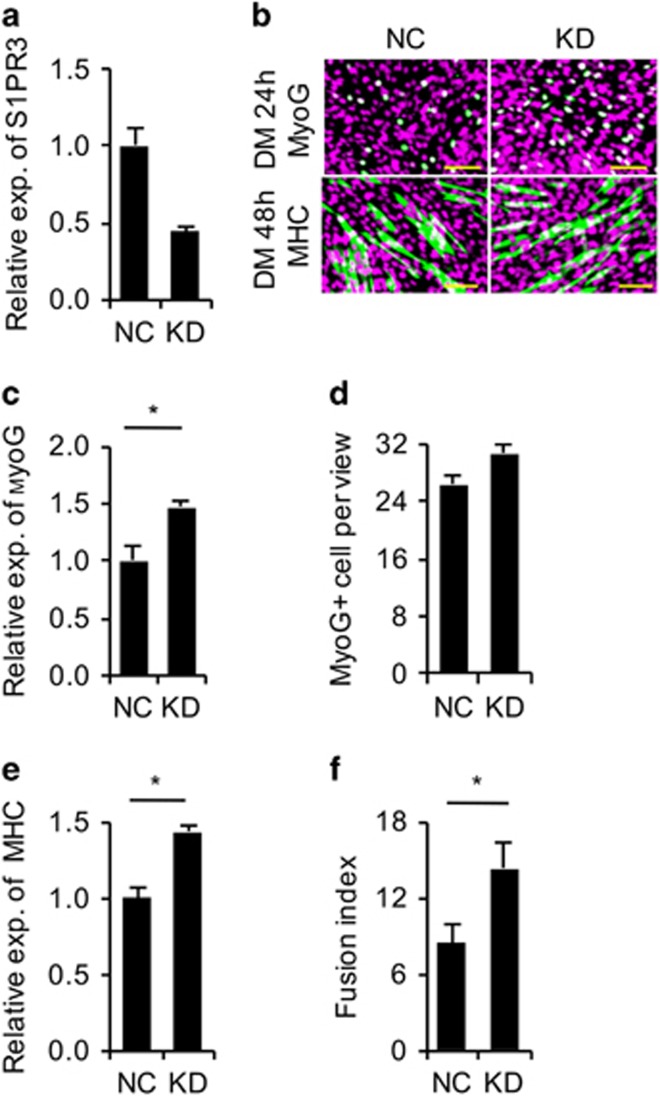
*S1PR3* knockdown promotes C2C12 cell differentiation. (**a**) Efficiency of siRNA-mediated *S1PR3* knockdown. Expression of *S1PR3* in C2C12 cells was analyzed by quantitative real-time polymerase chain reaction (qRT-PCR). The data were normalized using *GAPDH*. *S1PR3* expression levels were further normalized to the expression level of negative control (NC), defined as 1. (**b**) Immunostaining for MyoG (upper, green), MHC (lower, green) and DAPI (magenta) in C2C12 cells transfected with siRNAs against *S1PR3* or a scrambled NC sequence. Cells were cultured in DM for 24 h for MyoG staining and 48 h for MHC staining. DAPI staining indicates nuclei. Scale bars, 50 *μ*m. (**c**) Expression of *MyoG* in C2C12 cells described in panel b was analyzed by qRT-PCR. The data were normalized using *GAPDH*. *MyoG* expression levels were further normalized to the expression level of NC, defined as 1. (**d**) The number of MyoG^+^ cells in panel b was calculated. The data are representative of three independent experiments. For each experiment, 10 views were analyzed, and the data are presented as the number of MyoG^+^ cells per view. (**e**) Expression of *MHC* in C2C12 cells described in panel b was analyzed by qRT-PCR. The data were normalized using *GAPDH*. *MHC* expression levels were further normalized to the expression level of NC, defined as 1. (**f**) The fusion index for differentiated C2C12 cells described in panel b was calculated. The data are representative of three independent experiments; 10 views were analyzed for each experiment. Statistical analysis in panel f was performed with nonparametric tests. *P<0.05

**Figure 6:**
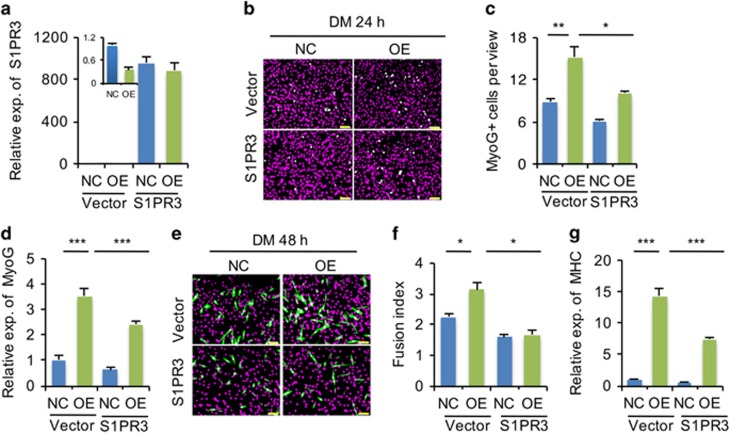
miR-127 enhances myogenic differentiation by targeting *S1PR3*. (**a**) Quantitative real-time polymerase chain reaction (qRT-PCR) analysis of *S1PR3* fold overexpression in miR-127 OE cells transiently transfected with an S1PR3 expression plasmid; cells transfected with empty vector served as a negative control (NC). The data were normalized using *GAPDH*. *S1PR3* expression levels were further normalized to the expression level of NC transfected with empty vector, defined as 1. (**b**) Immunostaining for MyoG (green) in miR-127 OE cells transiently transfected with an S1PR3 expression plasmid and NC cells transfected with empty vector. The cells were cultured in DM for 24 h. DAPI staining (magenta) indicates nuclei. Scale bars, 50 *μ*m. (**c**) Quantification of MyoG^+^ cells in panel b. The data are representative of three independent experiments. For each experiment, 10 representative views were analyzed, and data are presented as positive cell numbers per view. (**d**) Quantification of *MyoG* expression by qRT-PCR in cells described in panel b. The data were normalized using *GAPDH*. *MyoG* expression levels were further normalized to the expression level of NC transfected with empty vector, defined as 1. (**e**) Immunostaining for MHC (green) in miR-127 OE cells transiently transfected with an S1PR3 expression plasmid and NC cells transfected with empty vector (NC). The cells were cultured in DM for 48 h. DAPI staining (magenta) indicates nuclei. Scale bars, 50 *μ*m. (**f**) The fusion index, calculated as the percentage of the total nuclei present in multinucleated myotubes, was determined for MHC-stained cells presented in panel e. The data are representative of three independent experiments. For each experiment, 10 representative views were analyzed. (**g**) Quantification of *MHC* expression in cells indicated in panel (**e**). The data were normalized using *GAPDH*. *MHC* expression levels were further normalized to the expression level of NC transfected with empty vector, defined as 1. NC, C2C12 cells infected with control lentivirus; OE, C2C12 cells infected with miR-127-OE lentivirus. Values represent means±S.E. of at least three independent experiments. *P<0.05; **P<0.01; ***P<0.001

A previous study showed that skeletal muscle regeneration is enhanced in *S1PR3*-null mice.^33^ Interestingly, we found that the expression pattern of endogenous *S1PR3* during muscle regeneration (Figure 4h) was opposite that of miR-127 (Figure 4i), suggesting that miR-127 regulates myogenic differentiation by modulating S1PR3 function. Indeed, knocking down *S1PR3* (Figure 5a) promoted C2C12 cell differentiation (Figures 5b–f), a result consistent with the phenotype observed in *S1PR3*-null mice.^33^ To confirm directly the functional correlation between miR-127 and S1PR3, we overexpressed S1PR3 in C2C12 cells stably OE miR-127 (Figure 6a). MyoG^+^ cells were less numerous among C2C12 cells OE both miR-127 and S1PR3 (Figures 6b and c), and the level of MyoG mRNA in these cells was lower than that in cells stably OE only miR-127 (Figure 6d). Similar effects were also observed for MHC immunoreactivity (Figure 6e), fusion index (Figure 6f) and levels of *MHC* mRNA (Figure 6g), demonstrating that S1PR3 overexpression abolishes the miR-127-mediated enhancement of muscle cell differentiation. Collectively, these results demonstrate that miR-127 functionally regulates myogenic differentiation by targeting *S1PR3*.

### miR-127 attenuates the dystrophic phenotype of *mdx* mice

**Figure 7:**
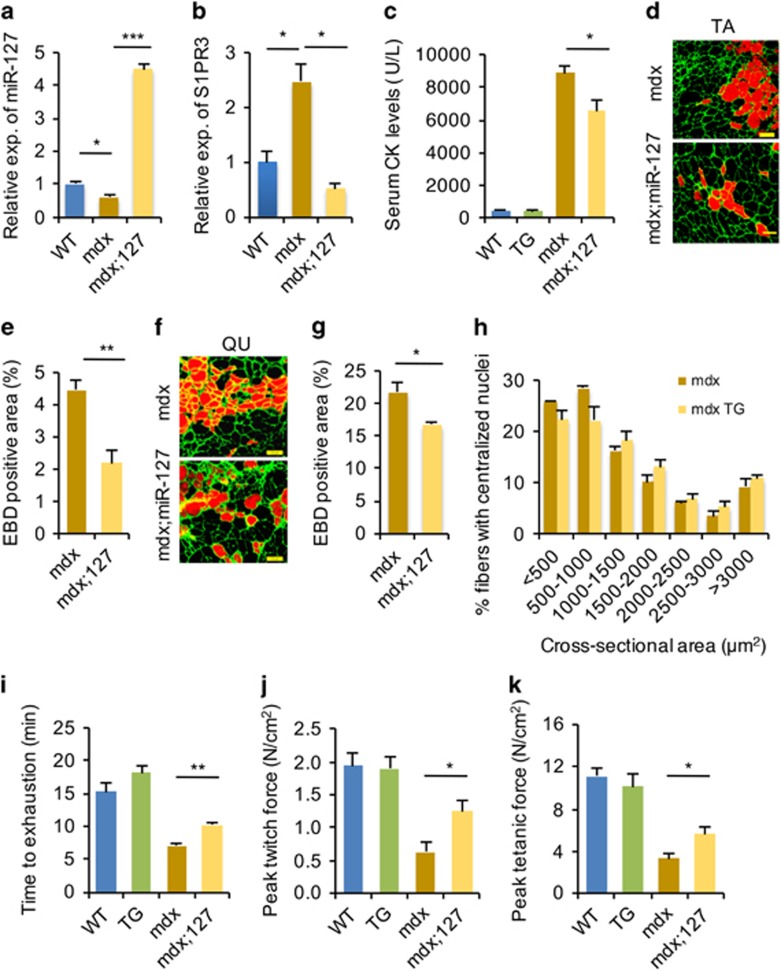
miR-127 ameliorates the dystrophic phenotype of *mdx* mice. (**a**) Fold overexpression of *miR-127-3p* in *mdx;miR-127* mice, determined by quantitative real-time polymerase chain reaction (qRT-PCR). The data were normalized using U6. *miR-127* expression levels were further normalized to the expression level of WT, defined as 1. (**b**) *S1PR3* mRNA levels in *mdx;miR-127* mice, determined by qRT-PCR. The data were normalized using GAPDH. *S1PR3* expression levels were further normalized to the expression level of WT, defined as 1. (**c**) Serum CK levels in WT, miR-127 TG, *mdx*, and *mdx;miR-127* mice at 3 month of age. (**d**) EBD uptake in TA muscles of *mdx* and *mdx;miR-127* mice at 3 month of age. EBD was detected as a red signal under a fluorescence microscope. Laminin immunostaining is shown in green. Scale bars, 100 *μ*m. (**e**) The percentages of EBD^+^ fiber areas in TA muscles from *mdx* and *mdx;miR-*127 mice. (**f**) Representative images of EBD^+^ areas in quadriceps muscles of *mdx* and *mdx;miR-127* mice at 3 month of age. EBD was detected as a red signal under a fluorescence microscope. Laminin immunostaining is shown in green. Scale bars, 100 *μ*m. (**g**) The percentages of EBD^+^ fiber areas in quadriceps (QU) muscles from *mdx* and *mdx;miR-127* mice. (**h**) Cross-sectional areas of myofibers with centralized nuclei in TA muscles from *mdx* and *mdx;miR-127* mice (*n*=5 mice per group). (**i**) Three-month-old WT, miR-127 TG, *mdx*, and *mdx;miR-127* mice were subjected to forced downhill running on a treadmill. Muscle performance was measured as total running time to exhaustion. (**j** and **k**) EDL muscles isolated from WT, miR-127 TG, *mdx*, and *mdx;miR-127* mice were electrically stimulated *in vitro* to elicit tetanic contractions. Maximal twitch force (**j**) and peak tetanic forces (**k**) were determined. Values are presented as means±S.E. (*n*=5 mice per genotype). *P<0.05; **P<0.01 and ***P<0.001

Given that miR-127 accelerates muscle regeneration in mice, we reasoned that miR-127 might reduce the muscular dystrophy phenotype in *mdx* mice. To this end, we generated *mdx* mice OE miR-127 (*mdx;miR-127*) and used them to assess the ability of miR-127 to alleviate symptoms of muscular dystrophy. miR-127 was overexpressed ~4.5-fold in the skeletal muscle of *mdx;miR-127* mice (Figure 7a). As a consequence, *S1PR3* mRNA levels were significantly reduced in the skeletal muscle of *mdx;miR-127* mice relative to those in *mdx* mice (Figure 7b).

Next, we further assessed the ability of miR-127 to attenuate the muscular dystrophy phenotype by measuring levels of serum creatine kinase (CK) and myofiber membrane permeability; the latter was examined by measuring uptake of Evans blue dye (EBD). Interestingly, levels of serum CK were significantly lower in *mdx;miR-127* mice than in *mdx* mice (Figure 7c). Consistent with this, EBD uptake was also significantly lower in both TA muscle (Figures 7d and e) and quadriceps (Figures 7f and g) of *mdx;miR-127* mice compared with *mdx* mice. Taken together, our data indicate that miR-127 overexpression significantly reduces the dystrophic muscle pathology in *mdx* mice by improving sarcolemmal integrity, despite the absence of obvious alterations in the cross-sectional area of myofibers in *mdx;miR-127* mice (Figure 7h).

Finally, we evaluated the physiological significance of miR-127 in *mdx;miR-127* mice by performing treadmill exercise experiments. Notably, the running time of *mdx;miR-127* mice was remarkably longer than that of *mdx* mice (Figure 7i). We also directly assessed physiological improvement in *mdx;miR-127* mice by measuring force generated by extensor digitorum longus (EDL) muscles from both *mdx* and *mdx;miR-127* mice. EDL muscles from *mdx;miR-127* mice exhibited a greater peak twitch force (Figure 7j) and a 1.5-fold increase in the peak tetanic force (Figure 7k) than those from *mdx* mice. Taken together, these findings show that miR-127 overexpression morphologically and functionally improves the dystrophic phenotype of *mdx* mice.

## Discussion

Recent studies have revealed the functional significance of miRNAs in regulating SC activation, proliferation, differentiation and fusion,^34^ but only a few miRNAs have been investigated for their involvement in controlling muscle regeneration *in vivo*. Very recently, Li *et al.*^27^ described a dynamic expression of miRNA-127-3p in proliferating and differentiating C2C12 cells as we reported here. Using transgenic mouse models in this study, we further demonstrate that miR-127 accelerates skeletal muscle regeneration in mice. Moreover, miR-127 overexpression significantly ameliorates muscular dystrophy symptoms in *mdx* mice. These findings suggest that miR-127 may be a potential therapeutic target in the treatment of human muscular diseases.

Mechanistically, we further provide evidence that miR-127 enhances SC differentiation by directly targeting the gene encoding S1PR3, a G-protein-coupled receptor for the bioactive sphingolipid S1P (sphingosine-1-phosphate), which is an important regulator of skeletal muscle function.^35^ Nagata *et al.*^36^ have shown that S1P mediates SC activation by inducing their entry into the cell cycle; thus, inhibition of S1P production following muscle damage greatly perturbs muscle regeneration. S1P signaling has been implicated in the regulation of muscle differentiation, homeostasis and SC function through interactions with its receptors, S1PR2 and S1PR3.^37^ S1PR2 is induced during myoblast differentiation, whereas S1PR3 is highly expressed in quiescent myogenic cells and is downregulated during entry into the cell cycle. Consistent with this differential expression pattern, S1PR2 has been shown to be a potent activator of myogenic differentiation,^38^ whereas S1PR3 appears to antagonize muscle differentiation. *S1PR3*-null mice exhibit enhanced muscle regeneration by virtue of increased SC proliferation and differentiation. Importantly, genetic deletion of *S1PR3* in the *mdx* mouse model (*S1PR3*^*−/*^*^−^;mdx*) significantly improves the muscle dystrophic phenotype.^33^

In this report, we identified *S1PR3* as a functional target of miR-127. In the context of CTX-induced muscle regeneration, miR-127 transgenic mice exhibited a phenotype similar to that observed in *S1PR3*-null mice. Notably, overexpression of miR-127 also considerably improved the muscular dystrophy phenotype in *mdx* mice by enhancing SC differentiation. Overexpression of S1PR3 significantly blocked miR-127-mediated myogenic differentiation. These data suggest the possibility that targeting S1P signaling is a promising strategy for treating muscular dystrophy. Intriguingly, a recent report demonstrated that genetically increasing S1P levels by manipulating S1P lyase (Sply) significantly improved dystrophic muscle phenotypes in *Drosophila*.^39^ Therefore, modulating the levels of the metabolite, S1P, or manipulating its receptor, S1PR3, by miR-127 would represent a potential therapeutic strategy for muscular dystrophy.

## Materials and Methods

### Mice and animal care

All animal procedures were approved by the Animal Ethics Committee of Peking Union Medical College (Beijing, China). All mice were maintained in a barrier facility with free access to water and standard rodent chow. C57BL/6 mice were obtained from Vital River Laboratories (Beijing, China). miR-127 transgenic mice in a C57BL/6 background were generated by the Model Animal Research Center of Nanjing University. Overexpression of miR-127 in the TG mice was driven by the *β*-actin promoter (pCAGGS). *mdx*;miR-127 mice were generated by breeding miR-127 transgenic male mice with homozygous *mdx/mdx* female mice. The gender- and age-matched littermates of the miR-127 TG and WT mice were used for all phenotypic analysis throughout the study.

### Muscle injury and regeneration

For muscle regeneration experiments, 8-week-old mice were injected with 30 *μ*l of 10 *μ*M CTX (Sigma, St. Louis, MO, USA) in PBS into the mid-belly of the right TA muscle. The left TA muscle of each mouse was injected with PBS as a negative control. Muscles were harvested 7.5 and 14 days after injection to assess the completion of regeneration and repair.

### C2C12 cell culture

Mouse C2C12 cells were cultured in growth medium consisting of Dulbecco's modified Eagle's medium (DMEM; Gibco, Life Technologies, Carlsbad, CA, USA) supplemented with 4.5 g/l glucose, 10% fetal bovine serum (FBS), 1% antibiotic/antimycotic and 1% gentamycin at 37 °C in a 5% CO_2_ atmosphere. For differentiation of C2C12 myoblasts into myotubes, cells were transferred to DMEM containing 2% horse serum (HS) and 1% penicillin/streptomycin, and then cultured for the indicated number of days. All cells were grown to ~80–90% confluence before induction of differentiation. C2C12 cell lines stably OE miR-127 were established by infection with lentivirus containing H1-miR-127-CMV-puromycin (Genechem, Shanghai, China). Mouse S1PR3 cDNA was amplified from mouse skeletal muscle cDNA by RT-PCR and then cloned into the pcDNA 3.0 expression vector (pcDNA 3.0-S1PR3). To overexpress S1PR3, miR-127-stable cell or control cells were transfected with 1.6 ng pcDNA 3.0-S1PR3 plasmids per 12-plate well by using the FuGene HD transfection reagent (Roche, Basel, Switzerland).

### siRNA duplex and transfection

A small interfering RNA (siRNA) duplex with the sense and antisense sequences, 5′-GGACCGUAGUGAUUGUGGUGAGUGU-3′ and 5′-ACACUCACCACAAUCACUACGGUCC-3′, respectively, targeting nucleotides 1199–1223 (5′-GGACCGTAGTGATTGTGGTGAGTGT-3′) in the coding region of mouse S1PR3 cDNA was used to knockdown S1PR3. Cells were plated at a density of 2 × 10^4^ cells per cm^2^, grown for 12 h, and transfected with 100 nM siRNA duplexes or a control siRNA using the FuGene HD transfection reagent (Roche) according to the manufacturer's recommendations.

### Isolation and culture of primary myoblasts

Hindlimb skeletal muscles were minced and digested with a mixture of type I collagenase and dispase B (Roche Applied Science, Basel, Switzerland). The obtained cells were filtered, centrifuged, and cultured in growth medium (F-10 Ham's medium supplemented with 20% FBS, 4 ng/ml basic fibroblast growth factor, and 1% penicillin–streptomycin) on collagen-coated cell culture plates at 37 °C, 5% CO_2_. The cell differentiation was induced in differentiation medium (DM) containing 2% HS and then cultured for 36 and 72 h, respectively.

### Northern blotting

Total RNA was extracted from the indicated tissues, separated by polyacrylamide gel electrophoresis (PAGE; 7 M urea) on 15% gels and transferred to a nylon membrane (N+ Amersham, New York, NY, USA). miR-127-3p probes were labeled with *γ*-^32^P-ATP using T4 DNA kinase (Fermentas, Thermo Scientific, Waltham, MA, USA). RNA blots were hybridized overnight in ULTRAhyb (Ambion, Thermo Scientific, Waltham, MA, USA) at 68 °C, washed two times with saline-sodium citrate (SSC)/0.1% sodium dodecyl sulfate (SDS) washing buffer, and then subjected to two stringent washes (2 × 30 min) with 0.1 × SSC/0.1% SDS wash buffer at 68 °C. The resulting RNA blots were exposed to X-ray film at −80 °C.

### Western blot analysis

Muscle tissues and C2C12 cells, detached by scraping, and were lysed on ice in lysis buffer (50 mM Tris (pH 7.5), 150 mM NaCl, 0.5% Nonidet P-40, and protease inhibitor cocktail). Proteins in lysates were resolved by SDS-PAGE, and then immunoblotted using primary antibodies against MHC (MF20; Developmental Studies Hybridoma Bank (DSHB)), MyoG (F5D; DSHB), S1PR3 (Abcam, Cambridge, MA, USA) and GAPDH (glyceraldehyde 3-phosphate dehydrogenase; Millipore, Bedford, MA, USA). After washing with Tris-buffered saline containing 0.1% Tween-20 (TBST) for 30 min, membranes were incubated with horseradish peroxidase-conjugated secondary antibodies (Zhongshanjinqiao Corporation, Beijing, China) for 1 h at room temperature, and then washed with TBST for 30 min. Membranes were then incubated for 1 min at room temperature in Detection Solution (Thermo, Scientific, Waltham, MA, USA), and subsequently exposed to X-ray film.

### Immunohistochemistry

C2C12 cells (2 × 10^4^ cells per cm^2^ in growth medium) were seeded in standard plastic 12-well culture plates. After cells reached 70–80% confluence, the medium was changed to DM, and cells were cultured for 24 or 48 h. Cells were then fixed with 4% formaldehyde, washed with PBS, permeabilized with 0.1% Triton X-100 at room temperature, blocked with 3% bovine serum albumin for 10 min, and incubated with primary antibodies (anti-F5D diluted 1:200 or anti-MF20 diluted 1:300) for 1 h. Cells were subsequently incubated with fluorescein isothiocyanate-conjugated. Anti-mouse secondary antibodies (Zhongshanjinqiao Corporation) for 30 min at room temperature, and then with 4′,6-diamidino-2-phenylindoledihydrochloride (DAPI) at room temperature. MyoG and MHC staining were imaged with an Olympus IX71 fluorescence microscope (WHN × /1022, America, Inc., Center Valley, PA, USA) equipped with DP2-BSW software (Olympus America, Inc., Center Valley, PA, USA). Ten representative views were taken for each sample in 12-well plates. For calculating MyoG+ cells, the MyoG signal and DAPI signal were overlaid with IPP program. The merged nuclei were characterized as MyoG+ cells. For measurement of fusion index, the total number of nuclei in each field of view and the total number of nuclei in multinucleated myotubes were counted using ImageJ (Bethesda, MD, USA), and the fusion indices was calculated from the ratio of these two numbers.

### Real-time RT-PCR analysis

Total RNA was extracted from cells using the TRIzol reagent (Invitrogen, Grand Island, NY, USA). The expression levels of mature miRNAs miR-127-3p were determined using the miRNA-specific TaqMan microRNA Assay Kit (Applied Biosystems, CA, USA), U6 was used as a normalizer. mRNA levels were assessed using the Fast Eva Green qPCR Master Mix (Bio-Rad, Hercules, CA, USA) and *GAPDH* was used as normalized control.

### Transfection and luciferase assays

Luciferase reporter assays were performed as described.^16^ HEK293 cells were cultured in DMEM (Gibco) containing 4.5 g/l glucose supplemented with 10% FBS and 1% penicillin–streptomycin. Cells plated in 24-well plates were co-transfected with pGL3-S1PR3-3′-UTR and miR-127 mimics (triplicates for each transfection). Empty pGL-3 vector was used as a negative control. A *Renilla* luciferase plasmid was co-transfected with the firefly luciferase reporter construct as a transfection control, and results are expressed as firefly luciferase activity relative to *Renilla* luciferase activity. Representative data from three independent experiments are presented in the study.

### Treadmill

Treadmill tests were performed using an Exer-3/6 apparatus (Columbus Instruments, San Diego, CA, USA). For acclimation, mice were subjected to treadmill running four times (every other day) before the test. Mice ran on the treadmill at a 20° downhill slope, starting at a speed of 16 cm/s. After 3 min, the speed was increased by 2 cm/s to a final speed of 36 cm/s. Exhaustion was defined as the point at which the animal was unable to remain on the treadmill despite electrical prodding.

### Force measurements

The mouse EDL muscle was dissected and mounted as described.^40^ Mice were killed by cervical dislocation immediately before harvesting muscle. The carcass was arranged in a supine position on a dissection tray with the leg pinned to the tray. Under a dissecting microscope, the skin was pulled back, the fascia was carefully opened, and the tibialis was peeled from the ankle upwards to expose the EDL. The EDL was removed, preserving as much tendon as possible on each end, and placed in a Petri dish containing lactated Ringer's solution. Both muscle tendons were tied with sutures, which were used to extend the EDL between clamps of a myograph and secure the tendons of the EDL muscle between the clamps. EDL muscles were constantly immersed in a physiological saline solution (118.5 mM NaCl, 4.7 mM KCl, 2.4 mM CaCl_2_, 3.1 mM MgCl_2_, 25 mM NaHCO_3_, 2 mM NaH_2_PO_4_, and 5.5 mM d-glucose). All solutions were continuously bubbled with 95% O_2_/5% CO_2_ (vol/vol) and were maintained at pH 7.4. Contractions were elicited by passing a current between two platinum electrodes located on opposite sides of the muscle. A twitch contraction was elicited with a single 0.3-ms square pulse of 10 V (supramaximal voltage), whereas a tetanic contraction was elicited with a 200-ms train of the same pulse at a frequency of 200 Hz. Contractions were elicited every 2 min during the experiment. Muscle length was adjusted to obtain the maximum tetanic force, and a 30-min equilibrium period was allowed before the acquisition of force-frequency measurements. Force was measured with a dual-mode muscle lever system (X88 stimulator; Grass, OR, USA) and digitized at 5 kHz using an analog-digital board (Grass). Peak twitch and tetanic force were calculated as the difference between the maximum force during contraction and the force measured 5 ms before the contraction.

### Statistical analysis

The results are presented as means±s.e. The statistical significance of differences between two means was calculated using nonparametric tests as indicated in the figure legend. Otherwise, the statistical analyses were performed with Student *t*-tests. A *P*-value<0.05 was considered to represent a statistically significant difference.
