# Supplementary material for: miR-127 enhances myogenic cell differentiation by targeting S1PR3
Source: Cell Death Dis. 2017 Mar 30;8(3):e2707–. doi: 10.1038/cddis.2017.128 (PMC5386531; doi:10.1038/cddis.2017.128)
Supplement: Supplementary Information [file cddis2017128x1.docx]

Supplementary information for:

**miR-127 Enhances Myogenic Cell Differentiation by Targeting S1PR3**

**Lili Zhai1, Rimao Wu1, Wanhong Han1, Yong Zhang1* and Dahai Zhu1***

Supplementary Figure S1

**
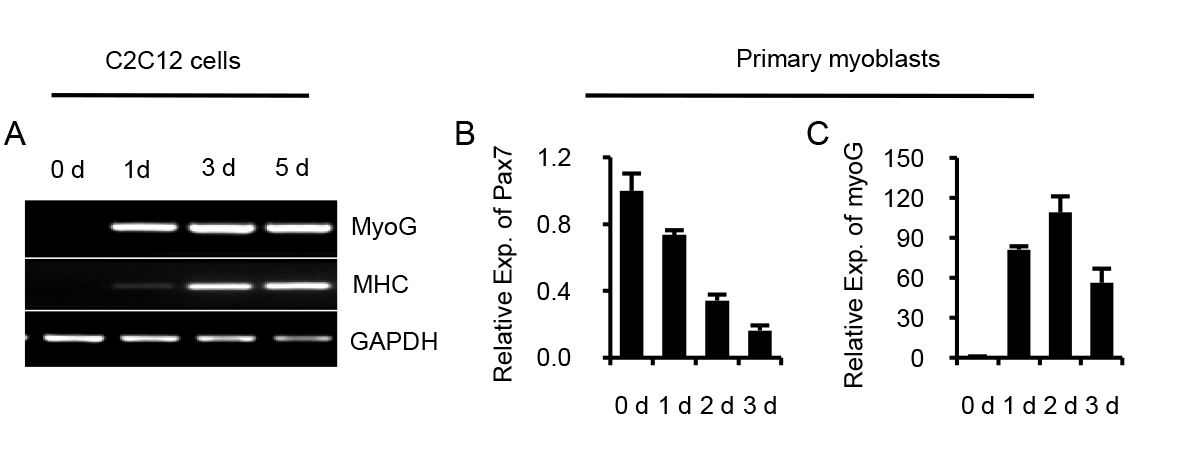
**

**Supplementary Figure S1.** **Differentiation of C2C12 cells and primary myoblasts were examined by measuring expression levels of differentiation markers MyoG or MHC during cell differentiation.** (A) Expression of MyoG and MHC in proliferating (0d in GM) and differentiating (1d, 3d and 5d in DM) C2C12 cells were analyzed by qRT-PCR. *GAPDH* served as equal loading control. (B-C) Expression of Pax7 (B) and MyoG (C) in proliferating (0d grown in GM) and differentiating (1d, 2d and 3d grown in DM) primary myoblasts were analyzed by qRT-PCR. *GAPDH* used as internal control. The data were further normalized to 0d, defined as 1. Values are means ± SE from triplicates experiments.
